# Organic Phosphorous and Calcium Source Induce the Synthesis of Yolk-Shell Structured Microspheres of Calcium Phosphate with High-Specific Surface Area: Application in HEL Adsorption

**DOI:** 10.1186/s11671-020-03298-w

**Published:** 2020-03-30

**Authors:** Xianshuo Cao, Guizhen Wang, Kai Wang, Lan Guo, Yang Cao, Xianying Cao, Yong Yang

**Affiliations:** 1 College of Life Science and Pharmacy, School of Materials Science and Engineering; State Key Laboratory of Marine Resource Utilization in South China Sea, College of Food Science and Engineering, Analytical and Testing Centre, Hainan University, Haikou, 570228 People’s Republic of China; 2 Department of Biochemistry and Molecular Biology, Hainan Medical College, Haikou, 571199 People’s Republic of China

**Keywords:** Yolk-shell, Calcium phosphate, Adsorption, Specific surface area

## Abstract

Yolk-shell-structured calcium phosphate microspheres have a great potential for medical applications due to their excellent physicochemical properties and biocompatibility. However, developing a yolk-shell-structured calcium phosphate with high adsorption capability remains a challenge. Herein, a porous yolk-shell-structured microsphere (ATP-CG) of calcium phosphate with high-specific surface area [*S*_BET_ = 143 m^2^ g^−1^, which is approximately three times as high as that of ATP-CL microspheres synthesized by replacing calcium source with calcium l-lactate pentahydrate (CL)] was successfully synthesized by using adenosine 5'-triphosphate disodium salt (ATP) as the phosphorous source and calcium gluconate monohydrate (CG) as calcium source through a self-templating approache. The influences of molar ratio of Ca to P (Ca/P), hydrothermal temperature, and time on the morphology of ATP-CG microspheres were also investigated. It is found that the organic calcium source and organic phosphorous source play a vital role in the formation of yolk-shell structure. Furthermore, a batch of adsorption experiments were investigated to illuminate the adsorption mechanism of two kinds of yolk-shell-structured microspheres synthesized with different calcium sources. The results show that the adsorption capacity of ATP-CG microspheres (332 ± 36 mg/g) is about twice higher than that of ATP-CL microspheres (176 ± 33 mg/g). Moreover, the higher-specific surface area caused by the calcium source and unique surface chemical properties for ATP-CG microspheres play an important role in the improvement of HEL adsorption capability. The study indicates that the as-prepared yolk-shell-structured microsphere is promising for application in drug delivery fields and provides an effective approach for improving drug adsorption capability.

## Introduction

Calcium phosphate has gained considerable attention during the past few years due to its excellent biocompatibility [1], high-loading capacity, and delivery efficiency. Calcium phosphate-related biomaterials have been widely used in various biomedical fields, such as tissue engineering [2], bone repair [3], and drug delivery [4]. In order to extend the application range and improve the performance of calcium phosphate-based materials, various calcium phosphate materials with varieties of morphologies and microstructures including carbonated hydroxyapatite (HAp) microspheres [5], HAp microtubes [6], hollow HAp microspheres [7], mesoporous yolk@cage-shell nanospheres of amorphous calcium phosphate (ACP) [8] have been reported.

Among various morphologies, yolk-shell-structured microspheres have attracted more and more attentions, as they are not only frontier materials science but also show unique morphological features. In yolk-shell-structured microspheres, the void space between yolk core and shell can serve as a storage reservoir for various cargoes and the porous-structured shell can provide a diffusion pathway for guest molecules, which makes them have great potential for diverse applications including catalysis [9], lithium-ion batteries [10], photocatalyst [11], and biomedicine [12]. Traditionally, sacrificing template methods are the primary ones to prepare yolk-shell-structured microspheres [13, 14]. These template strategies have achieved great success in adjusting the structure and properties. However, these approaches present some disadvantages. For instance, tedious processing steps and surfactant or structure-directing reagents, which may be hazardous to human being’s health. Currently, the self-templating methods have been widely used in the research of yolk-shell-structured microspheres [15, 16]. Unlike the traditional templating approaches, the templates employed in self-templating approaches are not only the templates for forming the voids, but also the precursor of yolk-shell-structured microspheres. Thus, self-templating methods are convenient approaches to prepare yolk-shell-structured microspheres. However, the introduction of self-templating approaches to the synthesis of yolk-shell-structured calcium phosphate microspheres remains an interesting challenge.

Furthermore, calcium phosphate materials have been utilized to carry different types of cargoes such as proteins [17], DNA [18], and siRNA [19]. However, poor drug adsorption capability of calcium phosphate needs to be solved urgently. Generally, the approaches of drug molecules immobilizing over the surface of carrier depend on the surface properties containing surface potential [20], hydrophobicity/hydrophilicity [21], hydrogen bond [22], and specific surface area [23]. So, improving surface properties and specific surface area is a valid approach for ehancing drug adsorption capability of carrier.

Herein, we prepared a kind of porous yolk-shell-structured microspheres of calcium phosphate by using adenosine 5'-triphosphate disodium salt (ATP) as the phosphorous source and calcium gluconate monohydrate (CG) as the calcium source through self-templating approach. Without any addition of templating agent, the as-prepared yolk-shell-structured calcium phosphate microspheres display a particularly high-specific surface area. Furthermore, the hen egg lysozyme (HEL) adsorption behavior of ATP-CG microspheres was investigated in comparison with the ATP-CL microspheres prepared by replacing calcium source with calcium l-lactate pentahydrate (CL). The results reveal that the difference of specific surface area caused by the calcium source and surface chemical properties play a vital role in the improvement of HEL adsorption capability.

## Methods

### Materials

Adenosine 5'-triphosphate disodium salt (ATP) was obtained from Macklin Biochemical Co., Ltd (Shanghai, China). Calcium gluconate monohydrate (CG) and calcium (l)-lactate pentahydrate (CL) were acquired from Sangon Biotech Co., Ltd (Shanghai, China). The hen egg lysozyme (HEL, ~ 70000 U/mg) was purchased from Sigma-Aldrich (Taufkirchen, Germany).

### Synthesis and Characterization of ATP-CG and ATP-CL Yolk-Shell-Structured Microspheres

The ATP-CG yolk-shell-structured calcium phosphate microspheres were prepared as follows: In brief, 0.9 g of CG was dissolved in 20 mL of ultrapure water to form solution C at 60 °C and 0.11 g of ATP was dissolved in 5 mL of ultrapure water to form solution P. Then, solution C was cooled down to room temperature and mixed with solution P under vigorous stirring and the pH of the solution was adjusted by 2 M NaOH solution to 5. The final volume of the solution was 30 mL with the extra addition of ultrapure water and the molar ratio of Ca to P (Ca/P) was 3.3. The final solution was transferred into a microwave digestion system for microwave hydrothermal reaction and treated at 120 °C for 15 min. The resulting precipitates were collected by centrifugation (4500 rpm, 10 min), rinsed with ultrapure water and lyophilized for 48 h. The ATP-CL microspheres were prepared according to literature procedures [24].

The crystalline phase of the microspheres was characterized by X-ray diffraction (XRD, Cu K_α_ source, *λ* = 0.154). The morphology of microspheres was observed by scanning electron microscopy (SEM), transmission electron microscopy (TEM), and high-resolution TEM (HRTEM). The compositions of microspheres were studied by Fourier transform infrared spectrophotometer (FTIR). The specific surface area of microspheres was determined by Brunauer-Emmett-Teller (BET). Thermogravimetryanalysis (TGA) was employed to study the thermal properties of samples at a heating rate of 10 °C/min in nitrogen atmosphere.

### HEL Adsorption and Characterization

The HEL adsorption experiments of two kinds of microspheres were conducted as follows: the certain amounts yolk-shell microspheres (ATP-CG, Ca/P = 3.3, 120 °C, 15 min, and ATP-CL, Ca/P = 2.5, 120 °C, 30 min) were dispersed in the water with constant ultrasonic treatment for 10 min to form 1.5 mg/mL of microspheres suspension. Then, 0.5 mL of aqueous solutions that contain various concentrations of HEL were immediately added into 1 mL above suspension and the final concentrations of drug were 1–7.5 mg/mL. Each solution was shaken (200 rpm) at 37 °C for 6 h. Later, the solutions were centrifuged and the amounts of HEL in the supernatants were measured by UV-vis spectrophotometer at 280 nm. The zeta potentials and compositions of microspheres before and after drug loading were characterized by zeta potential analyzer, FTIR spectrometer, and thermogravimetric analyzer (TGA, heating rate 10 °C min^−1^, nitrogen atmosphere).

### Adsorption Isotherm

In order to investigate adsorption behavior, Dubinin-Radushkevic isotherm (D-R) model was conducted in our study. The D-R model is based on the theory of micropore filling, which is used to describe the non-ideal sorption on a heterogeneous surface as well as distinguish the sorption mechanism (physical sorption or chemical sorption). The model is expressed by the following equation: where *Q*_eq_ is the adsorption capacity of adsorbent at equilibrium (mg/g), *C*_eq_ is the adsorbate concentration in the aqueous phase at equilibrium (mL/L). *Q*_m_ is the maximum adsorption capacity. *R* is gas constant, 8.314 J/(mol ∙ k). *T* is absolute temperature. *E* represents the mean free energy for estimating the type of adsorption. If the *E* value is below 8 kJ/mol, the adsorption type can be explained by physical adsorption, between 8 and 16 kJ/mol, the adsorption type belongs to ion exchange and greater than 16 kJ/mol, the adsorption type can be described by chemical adsorption.

1 $$ {Q}_{\mathrm{eq}}={Q}_m\exp \left(-{K}_{\mathrm{DR}}\ {\varepsilon}^2\right) $$

2 $$ \varepsilon = \text{RT1n}(1+\frac{1}{{C}_\text{eq}}) $$

3 $$ \mathrm{E}=\frac{1}{\sqrt{K_{\mathrm{DR}}}} $$

### Statistical Analysis of Drug Adsorption

Data were presented as mean ± standard deviation (SD) value. Significant differences (*p* < 0.05) were statistically calculated among different groups using the one-way ANOVA. All the experiments were carried out in triplicate and data was analyzed by using the DPS software.

## Results and Discussion

### Morphology and Chemical Characterization of Microspheres

#### ATP-CG Yolk-Shell-Structured Microspheres

**Fig. 1:**
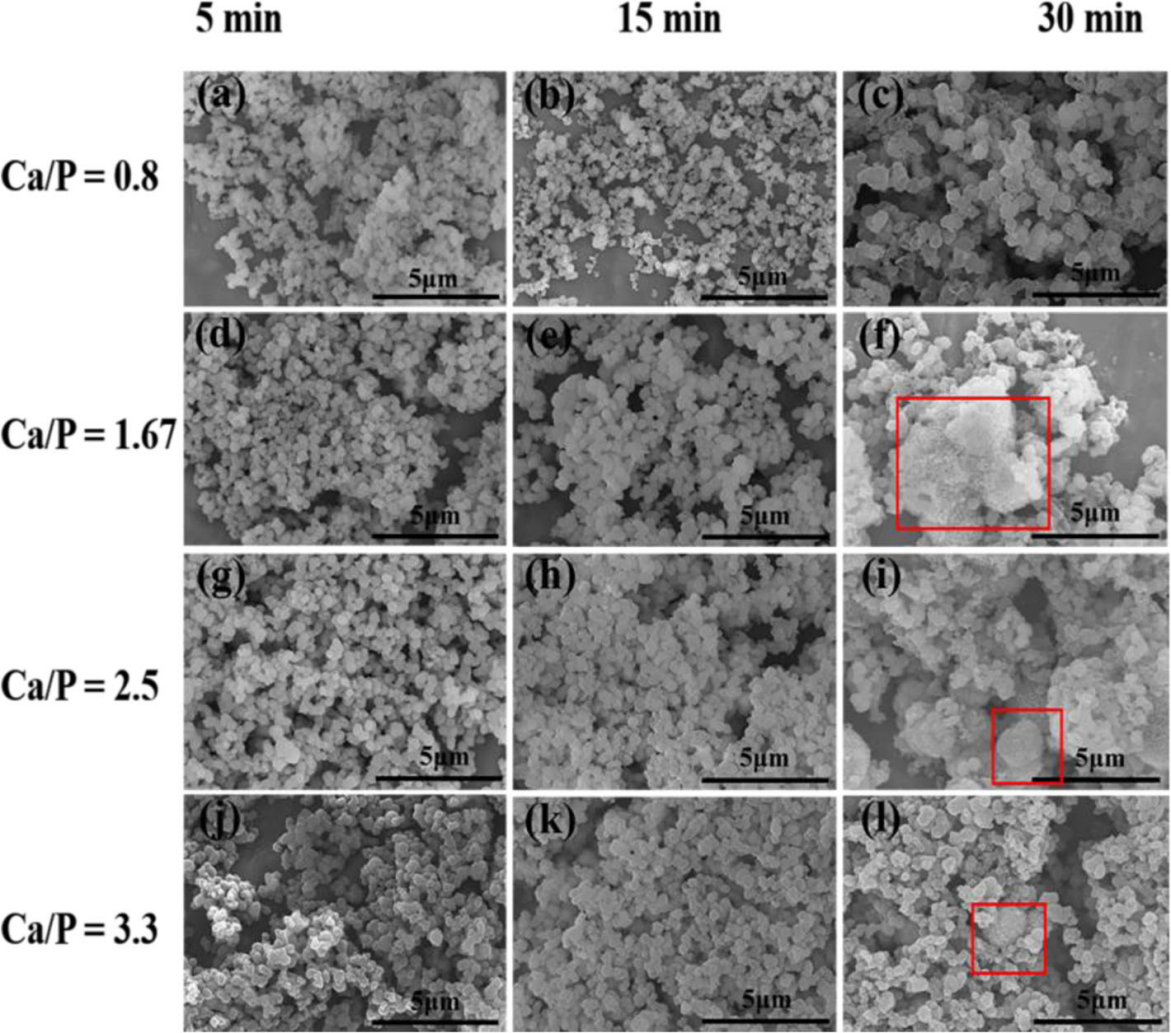
SEM images of ATP-CG microspheres prepared by microwave hydrothermal method at 120 °C

SEM images in Fig. 1 show the morphologies of various samples obtained under different reaction conditions. At *t* = 5 min or 15 min, all products are composed of uniform microspheres. However, when the hydrothermal time is further increased to 30 min, nanosheets self-assembled microspheres were formed (as shown in Fig. 1 f, i, l). Meanwhile, the effect of Ca/P on the morphology of products is also observed at *t* = 30 min. As the increase of Ca/P, the nanosheets self-assembled microspheres were gradually formed (as shown in Fig. 1 f, i, l). The formation of nanosheets self-assembled microspheres could be explained by the following reasons. Firstly, under the microwave hydrothermal process, ATP molecules could hydrolyze to form adenosine-based molecules including adenosine diphosphate (ADP), adenosine monophosphate (AMP) and adenosine, and simultaneously release phosphate ions (PO_4_^3−^). Meanwhile, CG molecules could hydrolyze to form gluconate and calcium ions (Ca^2+^). Then, phosphate ions would react with calcium ions to form primary ACP nuclei [25]. Then, the initial ACP nuclei grow and assemble to form ACP microspheres. Therefore, when hydrothermal time is further extended, the ATP and CG molecules in solution are further hydrolyzed and release more PO_4_^3−^ and Ca^2+^ ions, which causes the formation of nanosheets self-assembled microspheres through improving the supersaturation of system and the nucleation rate. In addition, by increasing the Ca/P, the local high concentration of Ca^2+^ also accelerates the morphology transformation of products in the same way as above. The above analysis indicates that the hydrothermal time and Ca/P have an important influence on the morphology of products.

**Fig. 2:**
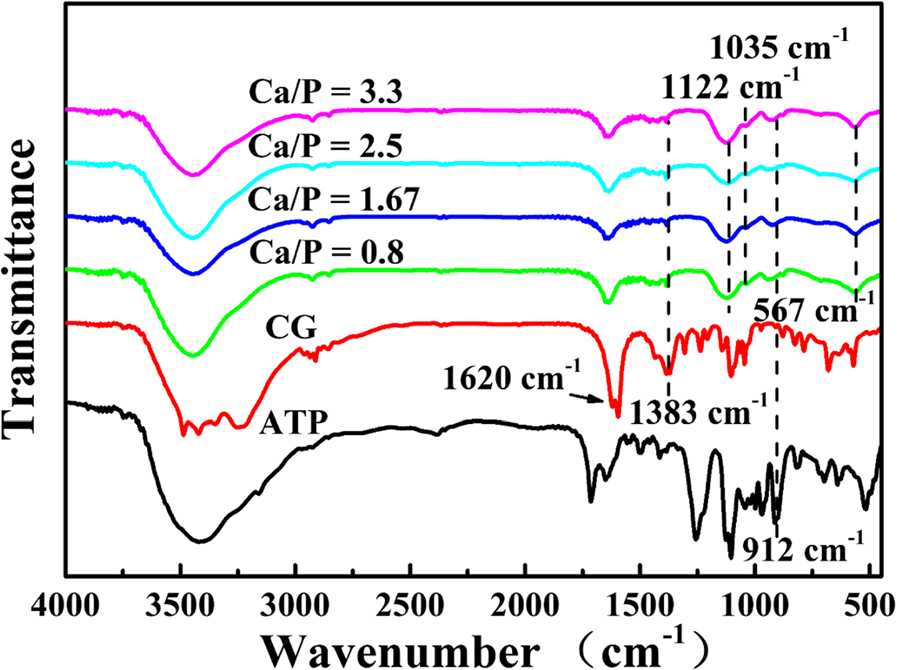
FTIR spectra of ATP-CG microspheres synthesized with various Ca/P at 120 °C for 15 min

Next, the FTIR spectra of microspheres synthesized with various Ca/P at 120 °C for 15 min are investigated (Fig. 2). The peaks at 1620 cm^−1^, 1383 cm^−1^, and 912 cm^−1^ attributed to the characteristic peaks of the C=O, C–O of CG and P–O groups of ATP [26], respectively, implying that unhydrolyzed CG and ATP molecules or their derivatives are absorbed on the surface of the microspheres. The faint characteristic peak of the PO_4_^3−^ from HAp is located at 1035 cm^−1^ [27] and the absorption peaks at 1122 cm^−1^ and 567 cm^−1^ are assigned to PO_4_^3−^ ions of ACP [28], indicating that the products are composed of ACP and HAp. The FTIR results suggest the calcium phosphate is succesfully prepared by using ATP as phosphorous source and CG as calcium source.

**Fig. 3:**
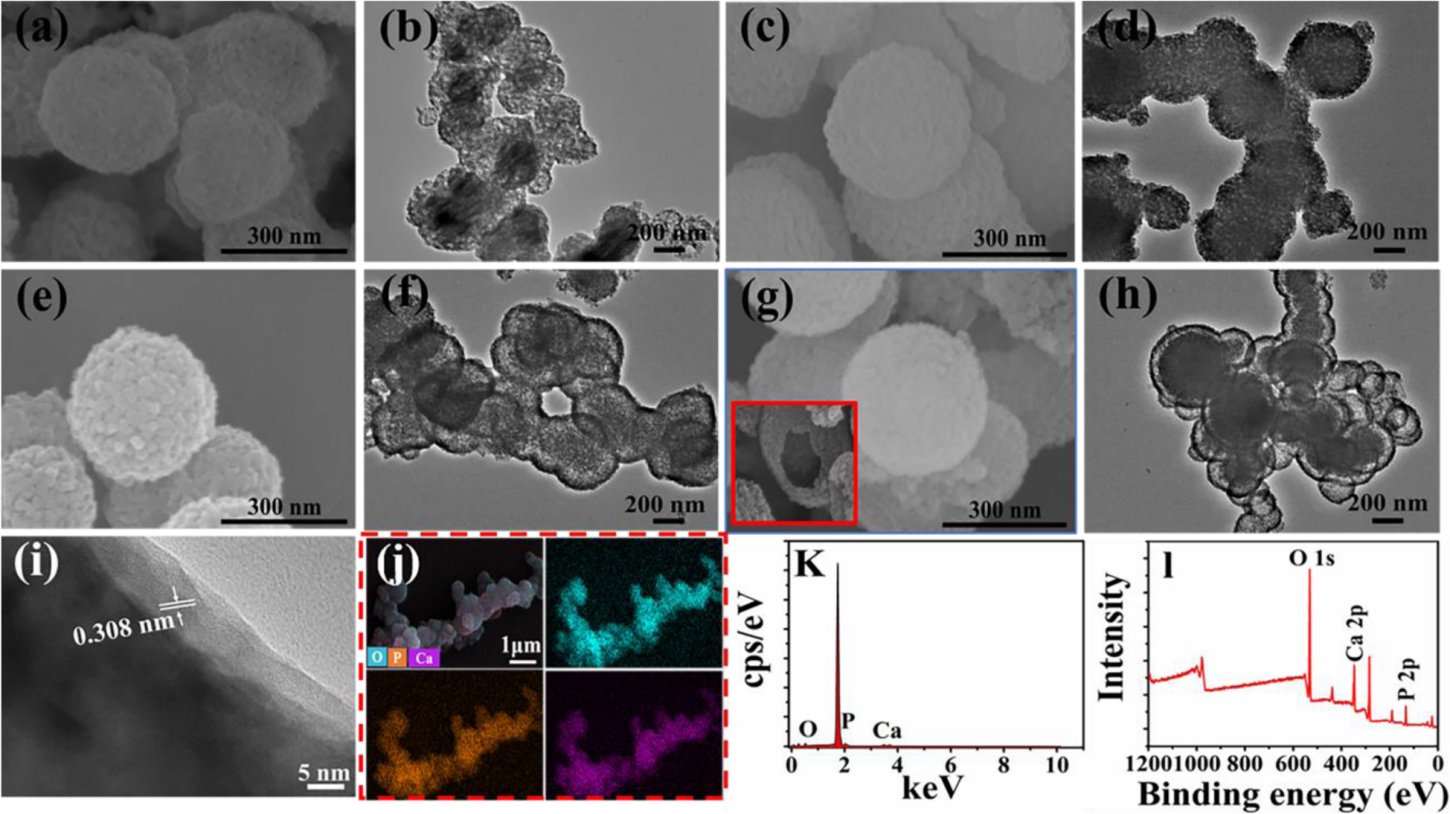
SEM and TEM images of ATP-CG microspheres synthesized with various of Ca/P. **a**, **b** Ca/P = 0.8. **c**, **d** Ca/P = 1.67. **e**, **f** Ca/P = 2.5. **g**, **h** Ca/P = 3.3. **i** HRTEM, **j** EDS-mapping, **k** EDS spectra, **l** XPS spectra of ATP-CG microspheres with Ca/P = 3.3

Furthermore, the SEM and TEM images of samples synthesized with various Ca/P through microwave hydrothermal method at 120 °C for 15 min are displayed in Fig. 3. When the Ca/P is 0.8 or 1.67, the samples consist of porous microspheres (Fig. 3b, d). When the Ca/P is 2.5, the morphology of products starts transforming to yolk-shell-structured microspheres (Fig. 3f). As the Ca/P further increases to 3.3, the products are entirely composed of yolk-shell-structured microspheres (Fig. 3h). Beyond that, some of broken spheres and the exposed cores of the yolk-shell microspheres (insert in Fig. 3g) are observed after mechanical fracturing, providing evidence of a hollow structure between yolk and shell. Based on the above observation, we tentatively propose the formation mechanism of yolk-shell-structured microspheres synthesized with various Ca/P. When the Ca/P is lower, the porous ACP microspheres are formed first, which is attributed to the inhibition effect of ATP and CG molecules or their derivatives adsorbed on the surface on the microspheres. Then, as the Ca/P is further increased, the metastable ACP will further grow, which is drived by the high supersaturation of system. Finally, the crystalline HAps are formed on the external surface, which is confirmed by the high-resolution TEM (HRTEM) image of microspheres in Fig. 3i (the interplanar distance of 0.308 nm can be indexed to (210) of HAp). As a result, the hollow structures between yolk and shell are generated due to the difference in volume or density between HAp and ACP [24]. The corresponding EDS mapping indicates that the Ca, P, and O elements are uniformly distributed throughout the microspheres. The EDS spectra in Fig. 3k and XPS spectrum in Fig. 3l reveal that the chemical elements of microspheres mainly include Ca, P, and O, which is consistent with the result of FTIR (Fig. 2).

**Fig. 4:**
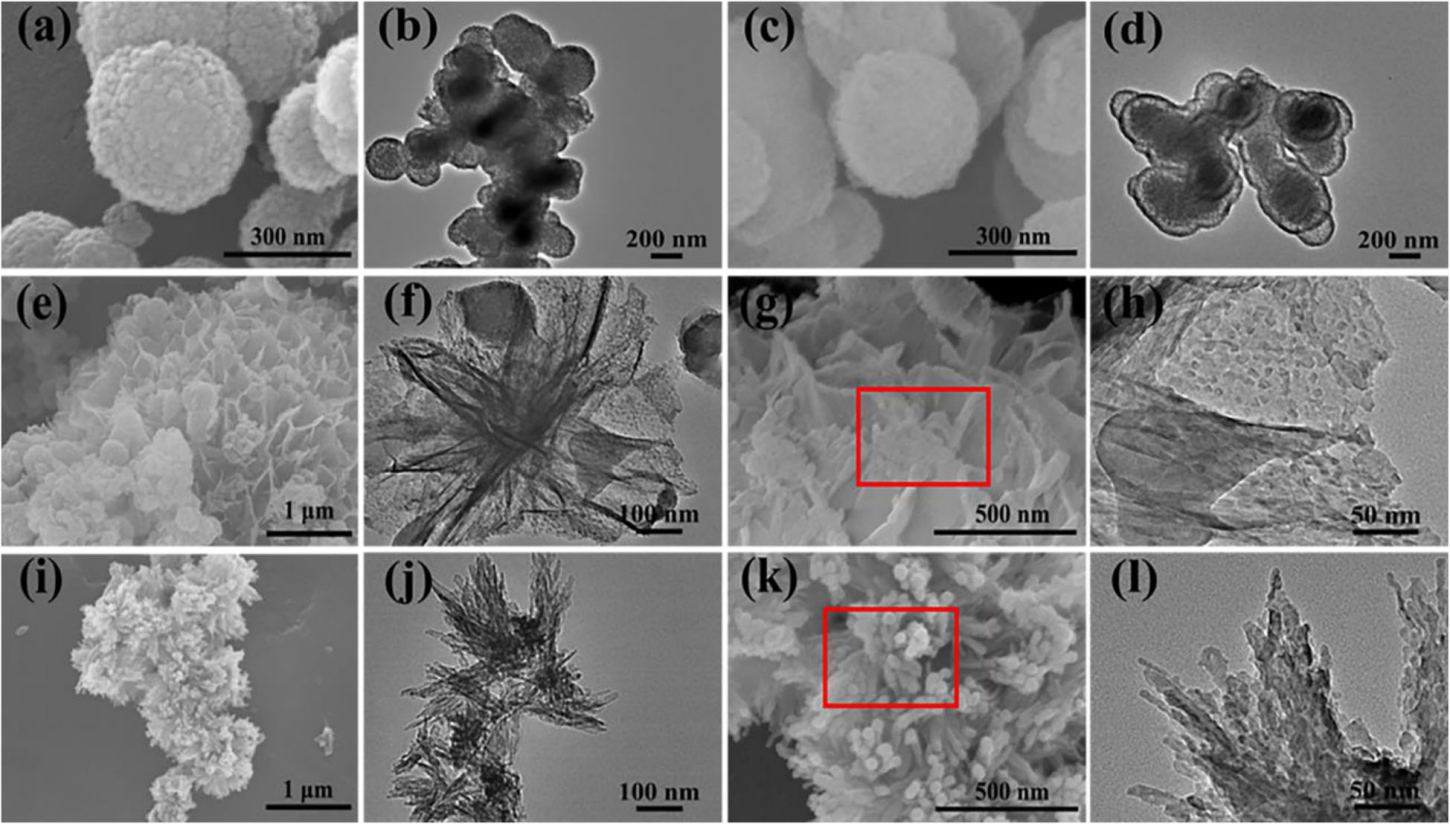
SEM and TEM images of ATP-CG microspheres synthesized with Ca/P = 3.3 under different experimental conditions. **a**, **b***T* = 120 °C, *t* = 5 min. **c**, **d***T* = 120 °C, *t* = 15 min. **e–h***T* = 120 °C, *t* = 60 min. **i–l***T* = 160 °C, *t* = 15 min

The impact of microwave-assisted hydrothermal time and temperature on the morphology of microspheres synthesized with Ca/P = 3.3 is further investigated. As shown in Fig. 4a-b, when the hydrothermal time is 5 min, the samples are composed of porous microspheres. As discussed above, when *t* = 15 min, the product is also composed of yolk-shell-structured microspheres (Fig. 4c-d). When hydrothermal time is extended to 60 min or temperature increased to 160 ^o^C, sheets or rods boundles are observed (Fig. 4e-l). The morphology transformation from porous to yolk-shell to sheet or rod is attributed to the further growth of ACP with the continuous hydrolysis of ATP and CG molecules in solution. Moreover, the hydrolysis of ATP and CG molecules or their derivatives adsorbed on the surface on the ACP microspheres also accelerates the growth of ACP. An interesting phenomenon emerged at 60 min or 160 ^o^C, these sheets or rods are also developed from ACP nanoparticles (as shown in red boxes), which is confirmed by the DTA analysis in Fig. S1. An exothermic peak at 650 °C is observed in the DTA curves [29, 30], which is attributed to the ACP crystallization. The exothermic peak gradually becomes weak with the increase of hydrothermal time or temperature, implying that the transformation of ACP in the products toward crystilline calcium phosphate.

**Fig. 5:**
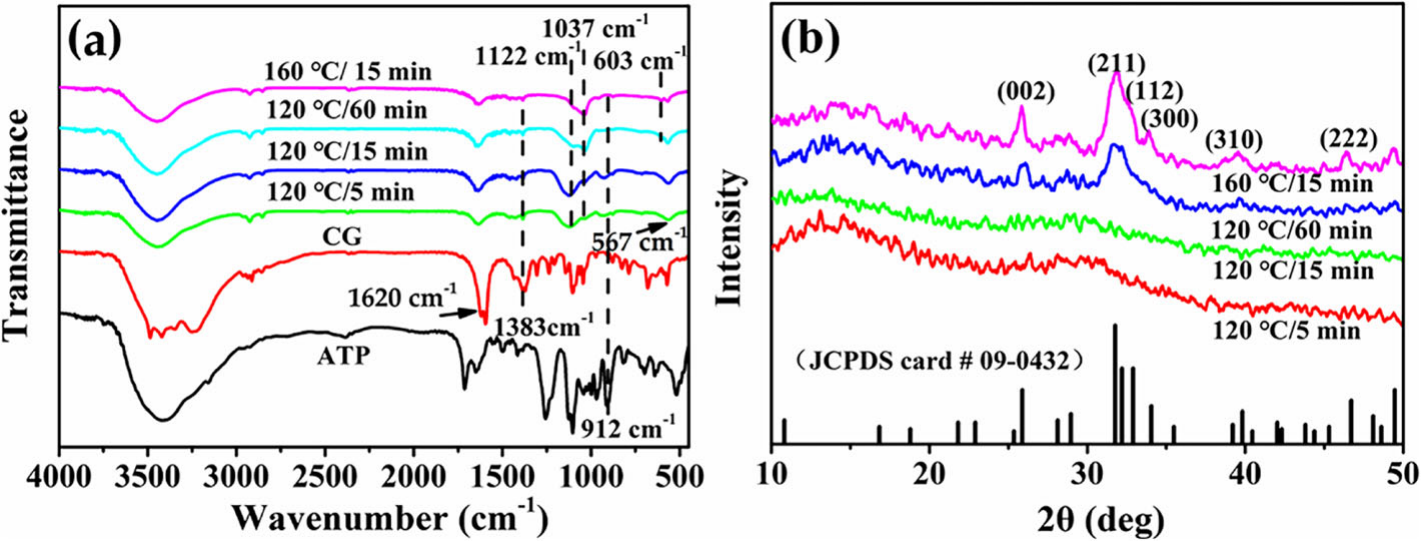
**a** FTIR spectra and **b** XRD patterns of ATP-CG microspheres synthesized with Ca/P = 3.3 under different experimental conditions

The chemical constitution and structure of samples synthesized with Ca/P = 3.3 under different hydrothermal time or temperature are investigated by the FTIR and XRD. As shown in Fig. 5a, the characteristic peaks of PO_4_^3−^ ions of HAp are located at 1037 cm^−1^ and 603 cm^−1^ [27]. The peak at 1122 cm^−1^ is assigned to characteristic peak of PO_4_^3−^ ions from ACP. The absorption peaks at 1620 cm^−1^ and 1383 cm^−1^ are attributed to characteristic peak of C=O and C–O groups from CG, respectively. The absorption peak at 912 cm^−1^ refers to the asymmetric P–O stretching vibration of ATP. By increasing hydrothermal time or temperature, the intensity of characteristic peaks of CG and ATP is gradually decreased, indicating the ATP and CG molecules or their derivatives adsorbed on the surface of microspheres are further hydrolyzed. Meanwhile, the intensity of characteristic peak of PO_4_^3−^ ions in HAp presents gradually increased trend with the decrease of intensity of ACP characteristic peak, illuminating the transformation of crystalline phase of products toward to HAp phase.

Figure 5b shows XRD patterns of different samples. A characteristic hump of amorphous phase at around 2*θ* = 30° of microspheres synthesized at 5 or 15 min is observed. However, when either hydrothermal time is extended to 60 min or temperature increased to 160 °C, the crystalline phase of microspheres thoroughly transforms to HAp, which could be indexed as the standard data (JDCPS no. 09-0432). The improvement in the relative intensity of (211), (300), and (002) lattice planes could further explain the increase in the crystallinity of products. Thus, the XRD and FTIR results further confirm the crystalline phase transformation of products with the increase in the hydrothermal temperature or time.

#### ATP-CL Yolk-Shell-Structured Microspheres

**Fig. 6:**
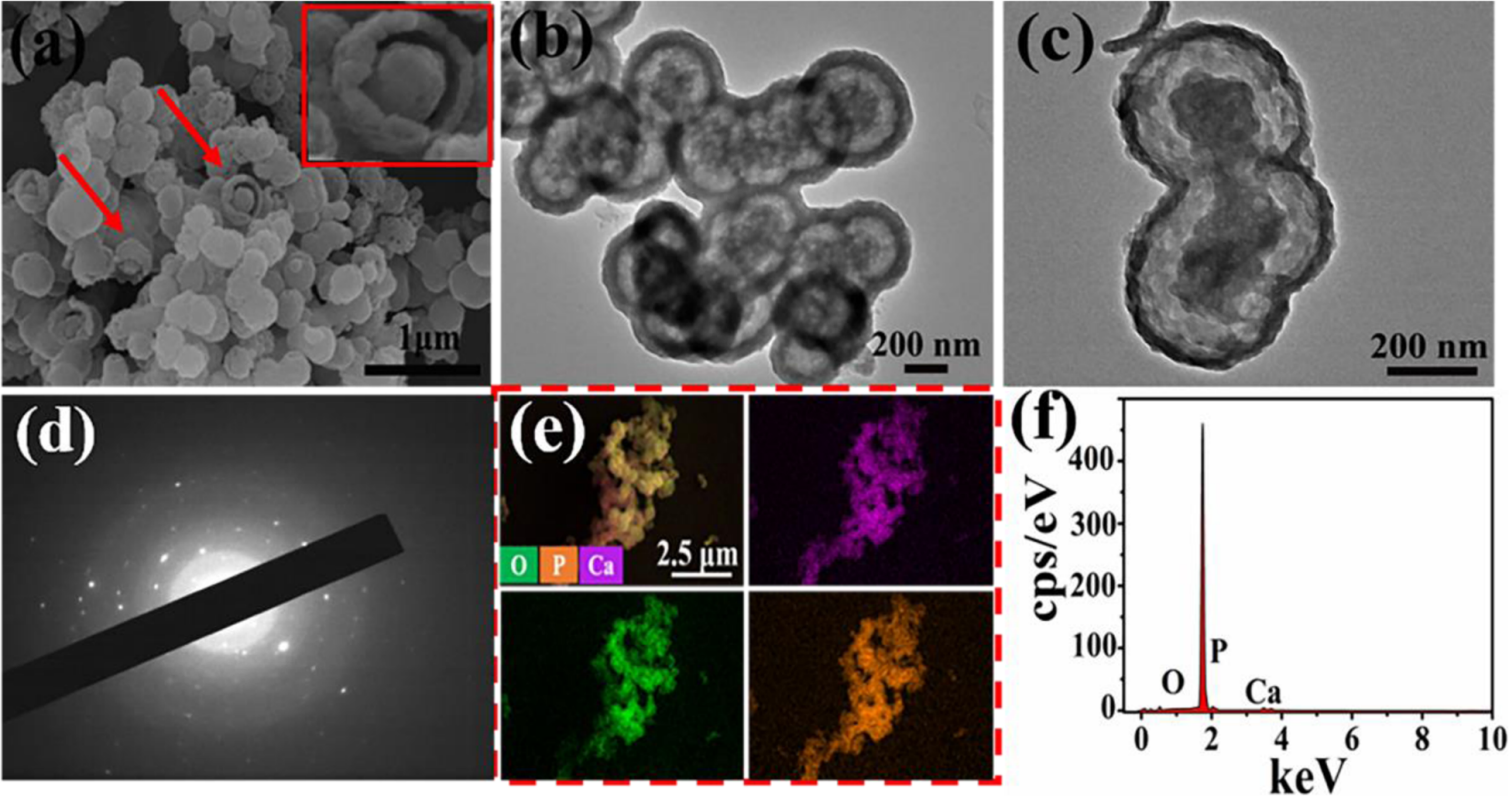
**a** SEM*.***b***,***c** TEM images*.***d***S*elected-area electron diffraction (SAED)*.***e** EDS-mapping and **f** EDS spectra of ATP-CL microspheres

In ordrer to compare drug adsorption behavior, the other yolk-shell-structured microspheres were prepared by using CL as organic calcium source through microwave hydrothermal method [24]. In terms of morphology, the samples still consist of yolk-shell-structured microspheres, which is verified by the broken spheres (insert in Fig. 6a) and TEM images (Fig. 6b, c). The result demonstrates that the change in calcium source have no significant effect on morphology of products. In addition, the selected-area electron diffraction (SAED) shows the discrete SAED spots (Fig. 6d), demonstrating that well-crystallized microspheres are obtained. In addition, the EDS-mapping exhibits the even distribution of Ca, P and O elements in microspheres (Fig. 6e). The corresponding EDS spectra also comfirms the presence of Ca, P, and O elemnets in microspheres (Fig. 6f), indicating that the as-prepared microspheres are calcium phosphate.

#### HEL Adsorption and Adsorption Mechanism of Microspheres

**Fig. 7:**
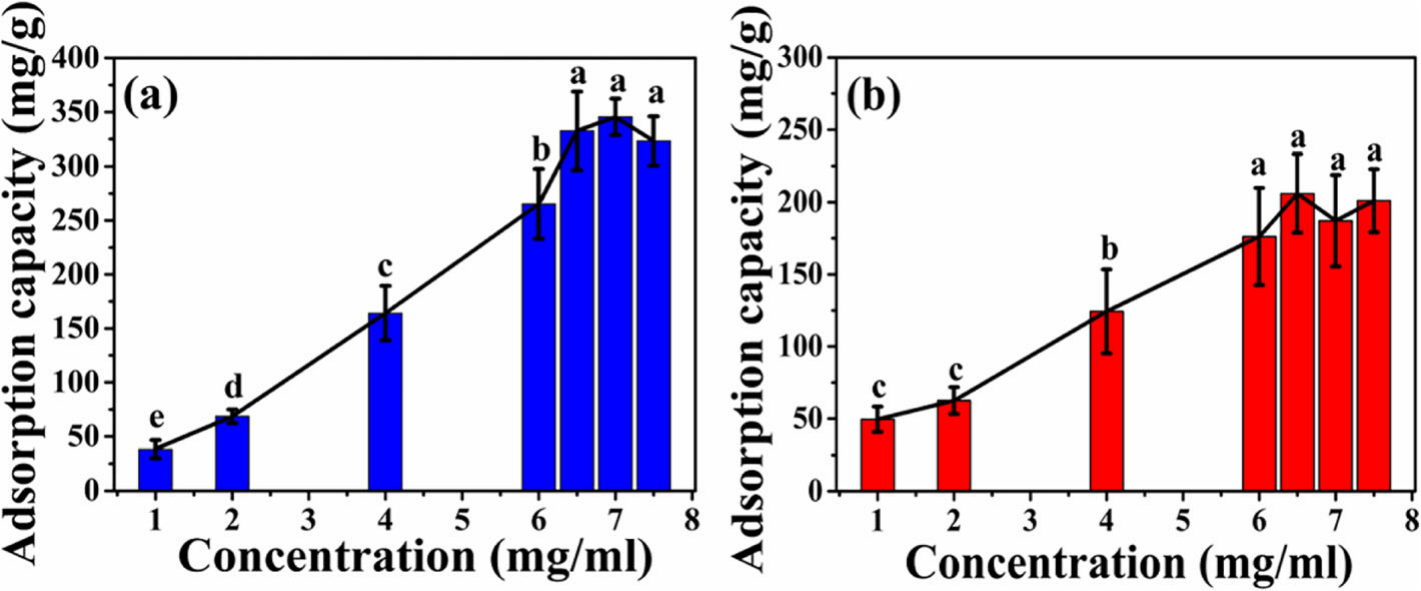
Adsorption curve of microspheres at different initial concentration of HEL. **a** ATP-CG microspheres. **b** ATP-CL microspheres

As shown in Fig. 7, the adsorption capacity of two kinds of microspheres increases with the increasing initial concentration of HEL. When the initial concentration of HEL increases to 6.5 mg/mL, the adsorption capacity of the ATP-CG microspheres reaches a plateau and the maximum adsorption capacity of microspheres is about 332 ± 36 mg/g (Fig. 7a), which is about twice higher than that of ATP-CL microspheres (176 ± 33 mg/g, 6 mg/mL, Fig. 7b).

**Fig. 8:**
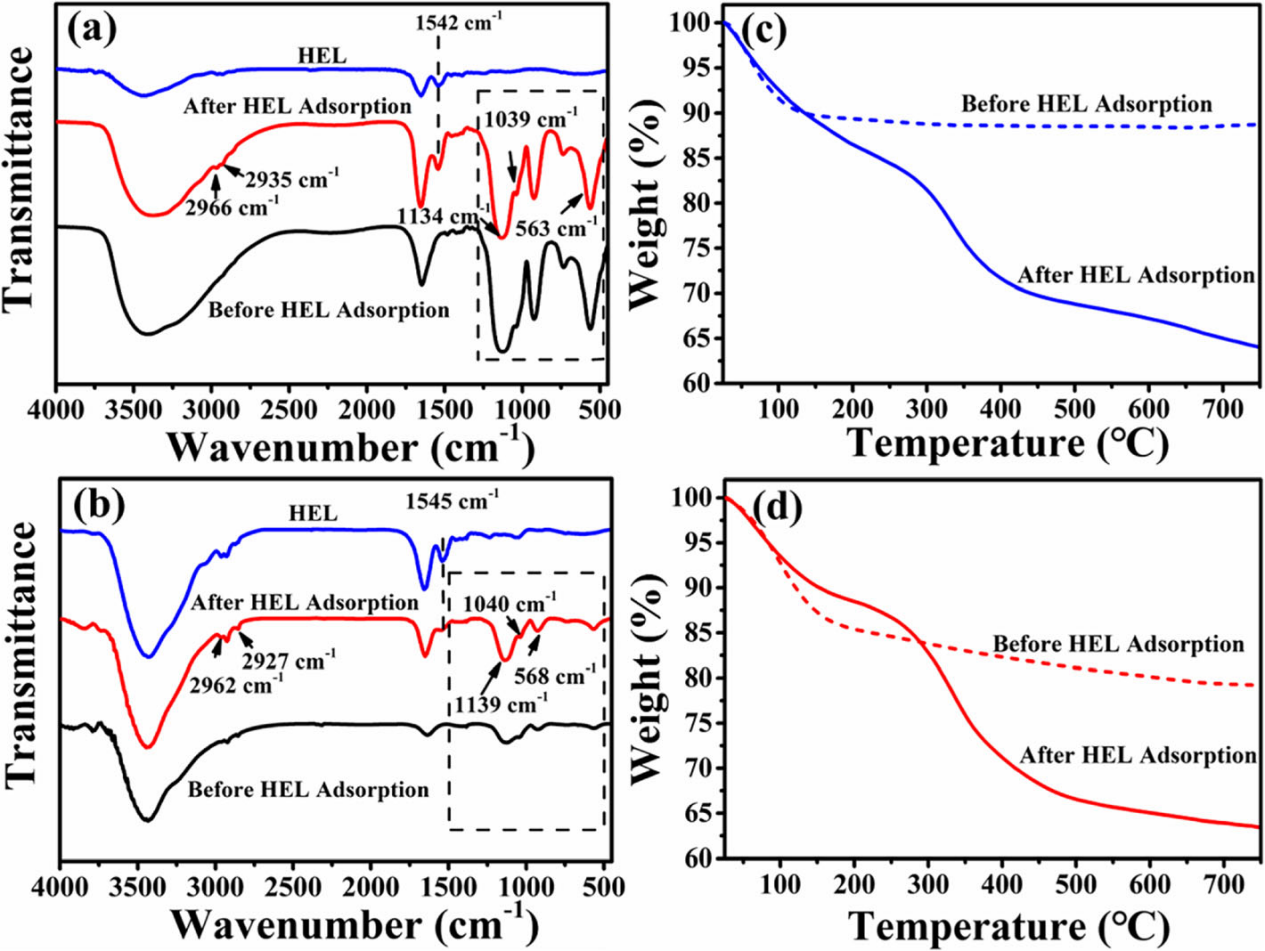
FT-IR spectra and TGA curves of microspheres before and after HEL adsorption*.***a** FTIR spectra and **c** TGA curves of ATP-CG microspheres, **b** FTIR spectra and **d** TGA curves of ATP-CL microspheres

The HEL adsorption result is further supported by the FTIR spectra and TG curves. As shown in Fig. 8a, b, the absorption peaks at 1134 cm^−1^ (1139 cm^−1^) and 563 cm^−1^ (568 cm^−1^) assigned to the characteristic peak PO_4_^3−^ ions of ACP and 1039 cm^−1^ (1040 cm^−1^) assigned to characteristic peak of PO_4_^3−^ ions of HAp are observed in the HEL-adsorbed microspheres, which indicates that the introduction of HEL in microspheres does not cause any significant change in the structure of microspheres. The adsorption peaks at 1542 cm^−1^ and 1545 cm^−1^ attributed to amide group of HEL are observed in HEL-adsorbed microspheres, confirming that HEL is successfully adsorbed on the microspheres. Meanwhile, the adsorption bands at 2966, 2962, 2935, and 2927 cm^−1^ originated from –CH_3_ and –CH_2_ groups of HEL are also detected in HEL-adsorbed microspheres, which further verifies the presence of HEL on the microspheres. The TGA curves display that the weight loss of ATP-CG microspheres before and after HEL adsorption is 11.3% and 36.7%, respectively (Fig. 8c). Therefore, the HEL adsorption capacity of ATP-CG microspheres is approximately 340 mg/g. However, a weight loss of 21.1% of ATP-CL is obtained before HEL adsorption and 37% appears on the HEL-adsorbed microspheres (Fig. 8d). So, the HEL adsorption capacity is 189 mg/g for ATP-CL microspheres. The TGA results are closed to the result from the Fig. 7.

**Fig. 9:**
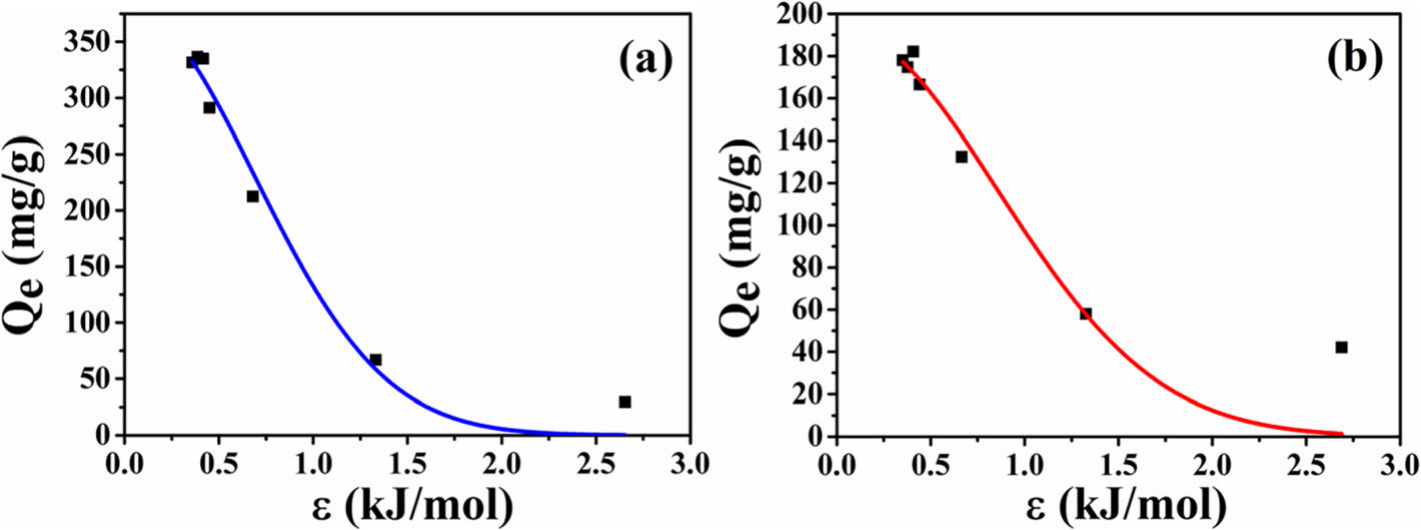
**a** Adsorption isotherms model of HEL on ATP-CG microspheres. **b** Adsorption isotherms model of HEL on ATP-CL microspheres

To investigate the cause of adsorption capacity difference between two kinds of microspheres, the equilibrium adsorption data of microspheres is further analyzed according to D-R isotherm model. The fitting curves are shown in Fig. 9 and fitting parameters are listed in Table 1, respectively. From the fitting results, the correlation coefficient from ATP-CG is higher than ATP-CL, suggesting D-R model is suitable for describing the drug adsorption behavior of ATP-CG microspheres. Since the *E* value is below 8 kJ/mol, the adsorption of HEL onto ATP-CG microspheres is physical sorption. The maximum capacity (*Q*_m_) of ATP-CG microspheres for HEL could reach as high as near 381 mg/g, which is close to result from Fig. 7a.

**Table 1:** Parameters of adsorption isotherms for microspheres

		ATP-CG	ATP-CL
D-R model	*Q*_m_ (mg/g)	381	192
	*R*^2^	97	89
	*E* (kJ/mol)	0.687	0.855

**Fig. 10:**
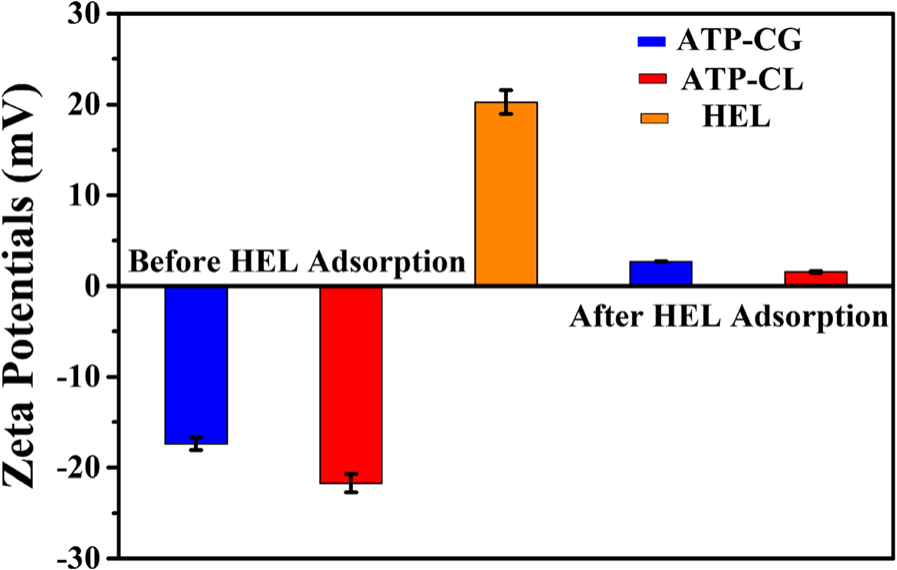
Zeta potentials of HEL and microspheres before and after HEL adsorption

Since the adsorption of HEL on the ATP-CG microspheres is physical sorption, the surface potential of microspheres is investigated. As shown in Fig. 10a, the zeta potential value of ATP-CG, ATP-CL microspheres and HEL in ultrapure water is – 17 mV, − 22 mV, and 20 mV, respectively. After HEL adsorption, the zeta potential value of ATP-CG and ATP-CL microspheres changes to 2.7 mV and 1.5 mV, respectively, indicating that the adsorption of HEL molecules onto the surface of microspheres through the attractive electrostatic force. However, the attractive electrostatic force is not the major cause of adsorption capacity difference between two kinds of microspheres, because there is no siginificant difference in zeta potential values (− 17 mV and – 22 mV) between microspheres.

**Fig. 11:**
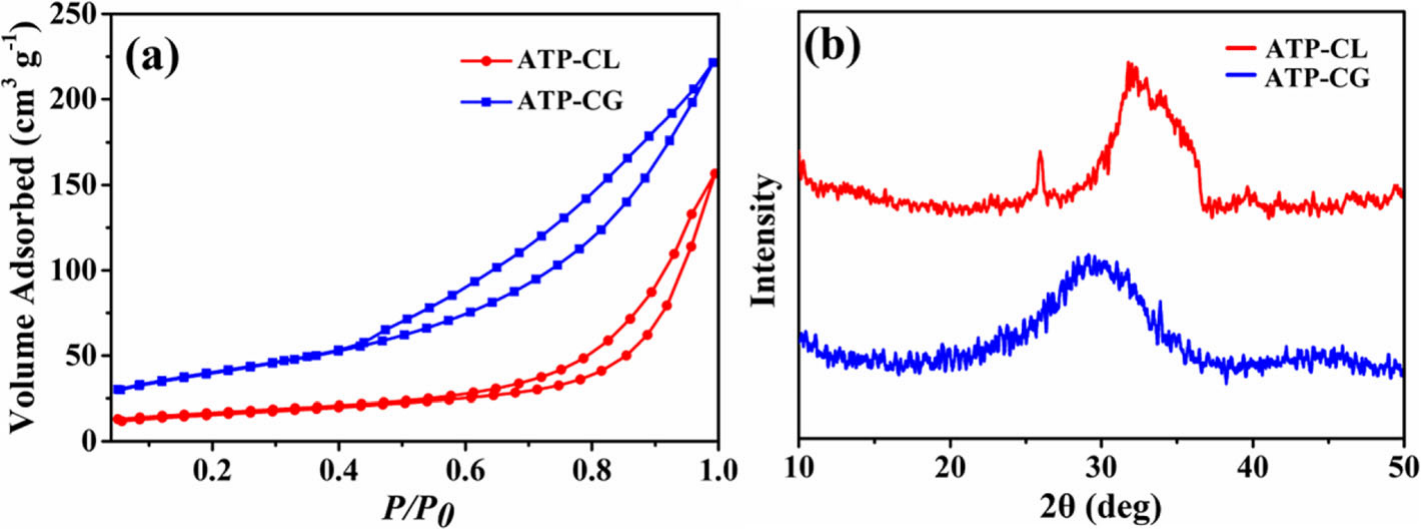
**a** Nitrogen adsorption-desorption isotherms. **b** XRD patterns of microspheres

Therefore, in order to further illuminate the reason causing absorption capacity difference between microspheres, the specific surface area of microspheres is investigated. As shown in Fig. 11a, the BET specific surface area (*S*_BET_) of ATP-CG microspheres is 143 m^2^ g^−1^, which is approximately three times as high as the ATP-CL microspheres (55 m^2^ g^−1^, Table 2). So, the speicific surface area can contribute to the absorption capacity difference between microspheres. Such high-specific surface area of ATP-CG microspheres is mainly attributed to the low crystallinity [31]. From Fig. 11b, ATP-CG microspheres exhibit lower crystallinity than that of ATP-CL microspheres. Moreover, the difference of crystallinity between ATP-CG and ATP-CL is chiefly due to the different synthesis conditions. Generally, the product crystallinity increases with the hydrolysis extent of reactants under certain pressure and temperature. Herein, the acidity of gluconic acid (pKa = 3.39) is higher than l-Lactic acid (pKa = 3.86), which would cause a slower hydrolysis rate and ultimately present a lower crystallinity. As a result, ATP-CG microspheres with a higher-specific surface area are obtained by chaging calcium source.

**Table 2:** Textural properties of microspheres

	ATP-CL	ATP-CG
*S*_BET_ (m^2^ g^−1^)	55	143
Pore size (nm)	16	8
Pore volume (cm^3^ g^−1^)	0.2	0.3

## Conclusions

The ATP-CG yolk-shell microspheres have been designed by using ATP as organic phosphorous source and CG as organic calcium source through a microwave-assisted hydrothermal method. The microspheres display a high-specific surface area and high adsorption capability. The influences of Ca/P, hydrothermal temperature, and time on the morphology and structure of microspheres were also investigated. The study indicates that organic phosphrous source and organic calcium source have a significant effect on the formation of yolk-shell-structured microspheres. Moreover, the hydrothermal conditions including Ca/P, hydrothermal, and temperature are responsible for the formation of yolk-shell microspheres. Furthermore, we find that the specific surface area and surface chemical properties such as surface potential are two key factors that affect adsorption capacity of microspheres by comparing the HEL adsorption behavior of two kinds of microspheres synthesized with different calcium source.

## Supplementary information

**Additional file 1: Figure S1.** DTA curves of ATP-CG microspheres synthesized with Ca/P = 3.3 under different experimental conditions.

## Data Availability

All data supporting the conclusions of this article are included within the article.

## Abbreviations

BET: Brunauer-Emmet-Teller measurements
FTIR: Fourier transform infrared spectroscopy
TEM: Transmission electron microscopy
XRD: X-ray diffraction
TGA: Thermogravimetry analysis
HRTEM: High-resolution TEM
SAED: Selected-area electron diffraction
