# Supplementary material for: Organic Phosphorous and Calcium Source Induce the Synthesis of Yolk-Shell Structured Microspheres of Calcium Phosphate with High-Specific Surface Area: Application in HEL Adsorption
Source: Nanoscale Res Lett. 2020 Mar 30;15:69. doi: 10.1186/s11671-020-03298-w (PMC7105591; doi:10.1186/s11671-020-03298-w)
Supplement: Supplementary file 1 — Additional file 1: Figure S1. DTA curves of ATP-CG microspheres synthesized with Ca/P = 3.3 under different experimental conditions. [file 11671_2020_3298_MOESM1_ESM.docx]

Organic Phosphorous and Calcium Source Induce the Synthesis of Yolk-shell Structured Microspheres of Calcium Phosphate with High Specific Surface Area: Application in HEL Adsorption

Xianshuo Cao^1,2,3^†, Guizhen Wang^5^†, Kai Wang^6^, Lan Guo^4^, Yang Cao^3^, Xianying Cao^3,4^* and Yong Yang^4^*

^1^ *College of Life Science and Pharmacy, Hainan University, Haikou 570228, China*

^2^ *School of Materials Science and Engineering, Hainan University, Haikou 570228, China*

^3^ *State Key Laboratory of Marine Resource Utilization in South China Sea, Hainan University, Haikou 570228, China*

^4^ *College of Food Science and Engineering, Hainan University, Haikou 570228, China*

^5^ *Analytical and Testing Centre, Hainnan University, Haikou 570228, China*

^6^ *Department of Biochemistry and Molecular Biology, Hainan Medical College, Haikou 571199, China*

1. **DTA curves of ATP-CG microspheres**





**Fig. S1** DTA curves of ATP-CG microspheres synthesized with Ca/P = 3.3 under different experimental conditions

The DTA curves of ATP-CG microspheres synthesized with Ca/P = 3.3 under different experimental conditions are displayed in the Fig. S1. The DTA curve of the sample prepared at 120 ℃ for 5 min shows an exothermic peak at 650 ℃, which is attributed the crystallization process of amorphous calcium phosphate (ACP). The exothermic peak gradually becomes weak with the increase of hydrothermal time or temperature, implying that the transformation of ACP in the products toward crystilline calcium phosphate. For the peoducts prepared at 160 ℃ for 15 min, the exothermic peak disappeared because theres is no ACP.
